# Asia Pacific Family Medicine: The rebirth of a not-so-young child

**DOI:** 10.1186/1447-056X-7-1

**Published:** 2008-09-29

**Authors:** Lyn Clearihan, TP Lam, Zorayda Leopando

**Affiliations:** 1 General Practice Training Program, Monash University/Victorian Metropolitan Alliance, Hawthorn, Victoria, Australia; 2 Family Medicine Unit, University of Hong Kong, Hong Kong; 3 Department of Family and Community Medicine, University of the Philippines Manila, Manila, Philippines

*Asia Pacific Family Medicine *was first published as a paper journal by Blackwell Publishing in 2002. At the time, we thought it was "a little like the birth of a child" [1]. Six years later, after some ups and downs, we are now moving to the open access web-based BioMed Central platform. This move to us, the Editors, is like the re-birth of a not-so-young child. Whilst the last 6 years have not been plain sailing for *Asia Pacific Family Medicine*, we believe there is still a strong need for the journal and it is anticipated that this new move will increase the exposure of the journal to a much wider audience. It is also an opportunity for us to build on what we have achieved so far and to take things further.

The objectives of *Asia Pacific Family Medicine*, despite the move, have remained unchanged. In 2002, we wanted a journal to provide a forum for the dissemination of high quality regional research and to enhance the standards of family medicine by focusing on best practice. In 2008, we are still wanting the same. This is because research forms the backbone of any medical discipline, this is perhaps even more so for a young discipline like family medicine. The only difference is that family medicine in the Asia Pacific region is much stronger than what it was six years ago. A most evident proof is that we are seeing many young and enthusiastic family doctors presenting excellent works in the Wonca Asia Pacific Regional conferences. However, many of these fine presentations are not being transformed into publications to reach a much wider audience. *Asia Pacific Family Medicine *is here to facilitate this. In fact, we welcome submissions from novice as well as experienced authors within and outside the Asia Pacific region.

With the professional support team at BioMed Central, *Asia Pacific Family Medicine *will be able to provide an efficient and effective publication channel for the large amount of family medicine research work that is done in the Wonca Asia Pacific region and around the world. The Editors are conscious of the publication charges in open access platforms like BioMed Central, which could pose a problem for some of our authors. We are however pleased that submissions from less developed countries in the region are provided waivers so that their good works are not being prevented from being published. In addition BioMed Central operates an institutional membership scheme, where by the article-processing charge is either wholly or partially covered by the researchers institution. For other authors of genuine needs, please feel free to contact us and we shall try to provide our best assistance.

The Editors would also like to acknowledge the strong support given to the journal by Wonca Asia Pacific and its 17 member organizations as well as the authors, reviewers and members of the Editorial Board in the past. *Asia Pacific Family Medicine *looks forward to their continued support.
